# Differential Cell Adhesion of Breast Cancer Stem Cells on Biomaterial Substrate with Nanotopographical Cues

**DOI:** 10.3390/jfb6020241

**Published:** 2015-04-21

**Authors:** Kenneth K.B. Tan, Christine S.Y. Giam, Ming Yi Leow, Ching Wan Chan, Evelyn K.F. Yim

**Affiliations:** 1 Mechanobiology Institute, National University of Singapore, T-Lab, #05-01, 5A Engineering Drive 1, Singapore 117411; E-Mail: mbiktkb@nus.edu.sg; 2 Department of Biomedical Engineering, National University of Singapore, EA-03-12, 9 Engineering Drive 1, Singapore 117575; E-Mails: zest228@gmail.com (C.S.Y.G.); leowmingyi@gmail.com (M.Y.L.); 3 Department of Surgery, Yong Loo Lin School of Medicine, National University of Singapore, NUHS Tower Block, Level 8, 1E Kent Ridge Road, Singapore 119228

**Keywords:** breast cancer, cancer stem cells, nanotopography, label-free isolation, cell adhesion

## Abstract

Cancer stem cells are speculated to have the capability of self-renewal and re-establishment of tumor heterogeneity, possibly involved in the potential relapse of cancer. CD44^+^CD24^−/low^ESA^+^ cells have been reported to possess tumorigenic properties, and these biomarkers are thought to be highly expressed in breast cancer stem cells. Cell behavior can be influenced by biomolecular and topographical cues in the natural microenvironment. We hypothesized that different cell populations in breast cancer tissue exhibit different adhesion characteristics on substrates with nanotopography. Adhesion characterizations were performed using human mammary epithelial cells (HMEC), breast cancer cell line MCF7 and primary invasive ductal carcinoma (IDC) cells obtained from patients’ samples, on micro- and nano-patterned poly-L-lactic acid (PLLA) films. Topography demonstrated a significant effect on cell adhesion, and the effect was cell type dependent. Cells showed elongation morphology on gratings. The CD44^+^CD24^−/low^ESA^+^ subpopulation in MCF7 and IDC cells showed preferential adhesion on 350-nm gratings. Flow cytometry analysis showed that 350-nm gratings captured a significantly higher percentage of CD44^+^CD24^−^ in MCF7. A slightly higher percentage of CD44^+^CD24^−/low^ESA^+^ was captured on the 350-nm gratings, although no significant difference was observed in the CD44^+^CD24^−^ESA^+^ in IDC cells across patterns. Taken together, the study demonstrated that the cancer stem cell subpopulation could be enriched using different nanopatterns. The enriched population could subsequently aid in the isolation and characterization of cancer stem cells.

## 1. Introduction

Despite recent improvements in breast cancer mortality, many patients relapse after initial response to conventional therapy and chemotherapy. Several alternative hypotheses have been proposed to explain this treatment failure and recurrence. Of particular interest is the cancer stem cell theory, which suggests that a small subpopulation of cells within tumors possess the unique ability to self-renew and generate the diverse cells that comprise the tumor [1]. Often referred to as cancer stem cells (CSCs), these cells are found to be resistant to therapy and, hence, may reinitiate tumor growth after treatment [1,2,3]. As such, isolation of this tumorigenic population from tumor tissues will be crucial for therapy development. Breast CSCs were first identified and isolated by Al-Hajj *et al.* [4]. When human breast tumors were propagated in non-obese diabetic severe combined immunodeficiency (NOD/SCID) mice, it was observed that as few as 100 of the CD44^+^CD24^−/low^ cells were able to give rise to new tumors. Thus, breast CSCs are postulated to have a CD44^+^CD24^−/low^ phenotype, strongly expressing the adhesion CD44 molecule, while very weakly expressing the adhesion CD24 molecule. Subsequent studies were conducted to validate Al-Hajj’s findings [5,6,7].

The CSC fractions in solid tumors have been observed to be highly impure, and thus, the reported frequencies for the same tumor types have varied enormously between different research groups [1]. Thus, more definitive markers are required to better characterize the CSCs. In the breast CSC field, it was observed from Abraham’s work that the proportion of CD44^+^CD24^−/low^ cells ranged from 0 to 40% in normal tissues [5]. Human breast cancer cell lines also differ quantitatively in the proportion of CD44^+^CD24^−^ cells [7]. The percentage of CD44^+^CD24^−^ cells within breast cancer cell lines was found to be uncorrelated with tumorigenicity [8]. In addition, studies have found that the epithelial specific antigen (ESA) cell surface molecule is overexpressed by the majority of human epithelial carcinomas, including breast carcinomas [8,9,10]. The tumorigenic activity of the CD44^+^CD24^−/low^ population was further enhanced when the CD44^+^CD24^−/low^ESA^+^ cell population was used [4]. Therefore, CD44^+^CD24^−/low^ESA^+^ is a potential breast CSC marker for further investigation of this group of breast cancer cells with tumorigenic properties. The methods used to isolate CSCs have generally revolved around the use of labels, such as selective expression of surface markers [1,4,11]. However, while this method enables the identification and isolation of a specific sub-group of CSC, the marker may not be able to serve as a universal marker for CSC. 

A second method of CSC isolation is the Hoechst dye exclusion. These cells are referred to as side population cells and have been found to exhibit CSC characteristics of tumorigenicity and chemotherapeutic drug resistance [12,13]. A third method based on the observation that CSCs lack 26S proteasome function has emerged [3,14]. Cells with reduced 26S proteasome activity express CSC markers and appear to be more tumorigenic than the control cells. They have also been demonstrated to characterize a sub-population of the CD44^+^CD24^−/low^ breast CSCs [15]. 

Label-free methods exploiting the differences in physical properties, such as cell size, density, cell adhesion and dielectric properties, are also being explored. Advantages conferred by label-free methods include less laborious and time-consuming procedures, as the preparation for staining before and after cell separation will not be needed. Nonetheless, one potential drawback of these methods is that these physical differences might be insufficient for accurate cell separation. Substrates act as intelligent surfaces capable of providing biochemical and topographical signals to guide cell adhesion, spreading, morphology, proliferation, and eventually, cell differentiation [16]. Numerous studies have demonstrated that cell adhesion behavior is significantly affected by surface nanotopography [17,18]. Cell adhesion can be increased or decreased by changing the material or geometry used to construct the surface structure [19]. Kwon and co-workers [19] used a nanotopographic substrate in a microfluidic approach to separate human breast cancer cells using cell adhesion as a physical marker. The breast cancer cell line (MCF7) and normal human breast epithelial cells (MCF10A) were observed to display different adhesion properties when cultured on substrates with different topographies. However, the cell adhesion analysis of the cell line mono-culture may differ from the heterogenicity of breast cancer tissues, and therefore, the cell adhesion properties of a heterogeneous population would need to be further investigated.

Thus, we hypothesize that different cell populations in the heterogeneous breast cancer tissue will respond differently to nanotopographical cues, potentially providing a means of enriching breast CSCs for isolation. The information can be used to develop a fast and easy method to isolate cancer stem cells, as well as different cell populations from cancer tissue, via a label-free method employing the differences in cell adhesion. Here, we investigated the adhesion characteristics of human mammary epithelial cells (HMEC), invasive breast cancer cell line MCF7 and invasive ductal carcinoma (IDC) primary breast tumor cells on nanopatterned poly-L-lactic acid (PLLA) films, as well as studying the effectiveness of these nanopatterns in preferential adhesion and isolation of breast CSCs. 

## 2. Results

### 2.1. Fabrication of Patterned Poly-L-Lactic Acid Films

**Figure 1:**
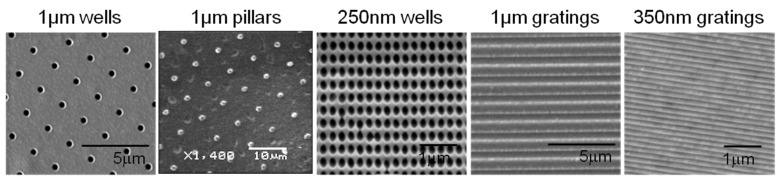
Scanning electron microscopic (SEM) images of poly-L-lactic acid (PLLA) patterned films with nano- and micro-structures.

Solvent casting was used to make the PLLA (3% w/v in chloroform) films from the poly(dimethylsiloxanes) (PDMS) masters with topographies of 250-nm wells, 1-µm wells, 350-nm gratings, 1-µm gratings and 1-µm pillars. PLLA solution was cast on the PDMS master. After drying overnight at room temperature and a brief vacuum drying to remove any residual solvent, the PLLA film was gently stripped off. The scanning electron micrographs (SEM) of the patterned PLLA films showed that the fabrication techniques used were able to replicate nanostructures accurately (Figure 1), verifying the fidelity of the replication. 

### 2.2. Influence of Surface Topography on Cell Morphology

**Figure 2:**
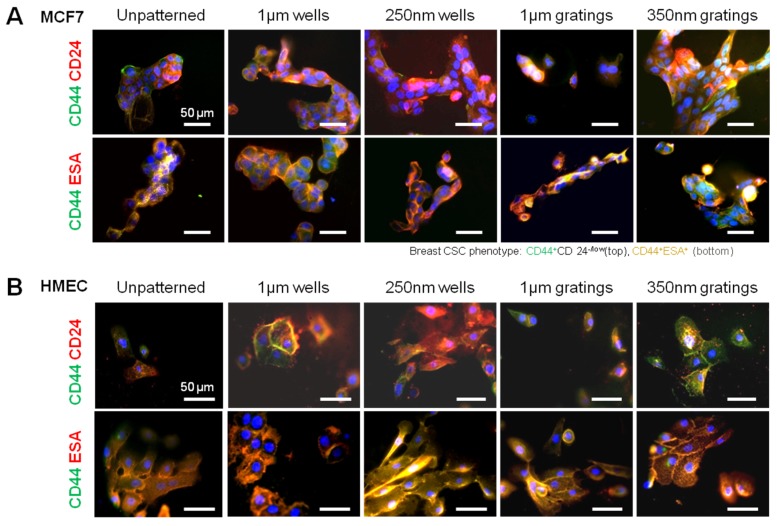
Immunofluorescence images of (**A**) MCF7 and (**B**) human mammary epithelial cells (HMEC) after 24 h of culture. The cells were stained for CD44 (green) and co-stained with either CD24 (red) or ESA (red), with DAPI as the counter-stain of nuclei (blue).

Immunofluorescence staining was used to examine the cell morphology and to assess the expression of CD44, CD24 and ESA qualitatively. MCF7 and HMEC have different cell morphologies when seeded on substrates with nanotopography after 24 h of culture (Figure 2). Both cell types attached well and in clusters onto the patterned PLLA films without pre-coating with extracellular matrix protein. While a round morphology was most commonly observed, MCF7 and HMEC were well spread on patterned PLLA compared to unpatterned PLLA (Supplementary Figure S1), and cells were slightly more spread on 1-µm wells, 250-nm wells and 1-µm gratings, although a significant difference was not observed among patterns. Cell elongation was observed on the grating patterns, showing that topography was interacting with cells and might affect differential adhesion within heterogeneous cell populations. 

In the heterogeneous population of MCF7 after 4 h of culture, the degree of surface marker expression varied across the population, but generally, the majority of HMECs were observed to be CD44^+^, CD24^+^ and ESA^+^ across all of the patterns. On the other hand, the expression of CD44, CD24 and ESA in MCF7 cells varied between different patterned samples (Supplementary Figure S2). The heterogeneity in the immunophenotype of the MCF7 cells after a 24-hour culture period was also clearly reflected in Figure 2. The differences in the subpopulations were further analyzed with cell counting and flow cytometry.

### 2.3. Differential Adhesion of MCF7 and HMEC on Patterned PLLA Films

**Figure 3:**
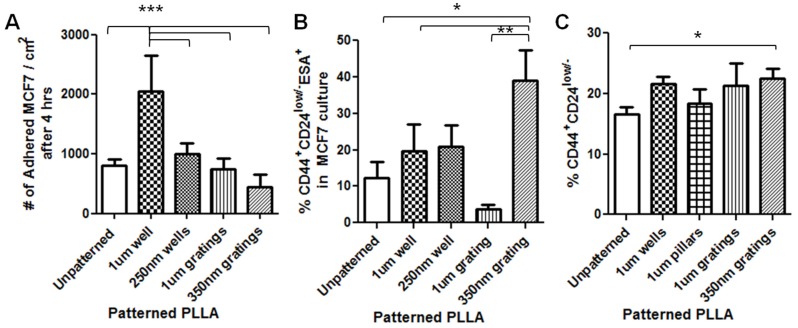
Effect of nanotopography on MCF7 cell adhesion 4 h after seeding on PLLA films. (**A**) Number of MCF7 attached on PLLA films, seeding density 16,000 cells/cm^2^. The 1-μm well has more attached cells, and the 350-nm grating has less attached cells (***P < 0.001, mean ± SD, *n* = 5); (**B**) MCF7 culture after 4 h showed that the 350-nm grating captured a significantly higher percentage of CD44^+^CD24^−/low^ESA^+^ cells (*P < 0.05, **P < 0.01, mean ± SD, *n* = 3); (**C**) Flow cytometry data showed that the 350-nm grating captured a higher percentage of CD44^+^CD24^−^ cells. The percentage of CD44^+^CD24^−^ cell population in MCF7 cells 4 h after seeding on PLLA films (*P < 0.05, mean ± SEM, *n* = 3).

**Figure 4:**
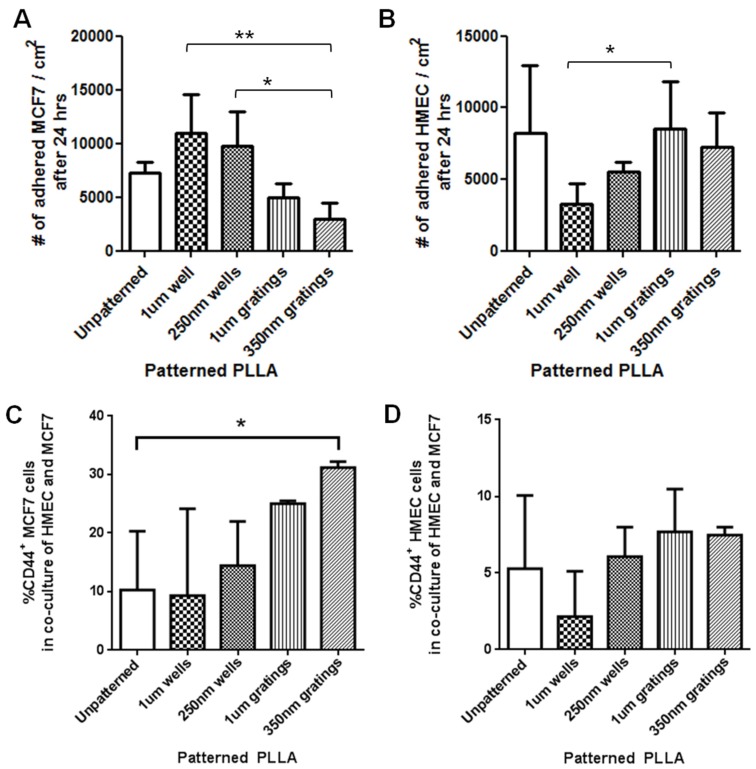
(**A**) Number of MCF7 attached 24 h after seeding on PLLA films, seeding density 16,000 cells/cm^2^ (*P < 0.05, **P < 0.01, mean ± SD, *n* = 5); (**B**) Number of HMEC attached 24 h after seeding on PLLA films, seeding density 16,000 cells/cm^2^ (*P < 0.05, mean ± SD, *n* = 5). Percentage of CD44^+^ (**C**) MCF7 and (**D**) HMECs from HMEC-MCF7 co-culture. The graph represents the percentage of CD44^+^ cells (in the CD44^+^/CellTracker Red^−^ region for MCF7 or the CellTracker Red^+^ region for HMEC) in an event count of at least 5000 after the 24-hour culture period on the PLLA films. Co-cultures of HMEC and MCF7 were seeded on PLLA films, and after 24 h of culture time, cells were fixed and stained for CD44.

The number of attached cells after a certain time point is a key parameter in determining the adhesion behavior of the cells. Cell adhesion is drastically influenced by the surface topography, thus affecting the number of cells that adhere to the substrate [20,21]. The number of attached cells on each of the PLLA films at the time points of 4 and 24 h is presented in Figure 3A and Figure 4A. 

Two different experimental setups were used to measure the number of adhered MCF7 cells on the PLLA film. In the first one, to assess the total number of adhered cells, cells were seeded on PLLA film; after the experimental time points (4 and 24 h), cells were detached enzymatically using trypsin/EDTA and counted using a hemocytometer. In another experimental setup to assess the percentage of CD44^+^CD24^−/low^ESA^+^ in the population, the cells and nuclei were fluorescently stained. Images were taken of random areas (at least five per sample) and counted.

After 4 h of culture of MCF7 cells on the various PLLA films, the 1-μm well had more attached cells compared to all samples and the control (unpatterned) (*P* < 0.001, mean ± SD, *n* = 5) (Figure 3A). Cell counting after 4 h of culture for CD44^+^CD24^−/low^ESA^+^ MCF7 cells showed that a higher number of CD44^+^CD24^−/low^ESA^+^ MCF7 cells were captured on the nanoscale gratings, as compared to the control and other micro-patterns (*P* < 0.05, *n* = 3) (Figure 3B). Flow cytometry analysis also showed that 350-nm gratings captured a significantly higher percentage of CD44^+^CD24^−^ than the unpatterned control (*P* < 0.05, *n* = 3). There was no significant difference in the percentage of CD44^+^CD24^−^ cell population in MCF7 across different patterns (Figure 3C).

After 24 h of culture on the various PLLA films, MCF7 cell counting showed that 1-µm wells and 250-nm wells had more attached cells than other samples (Figure 4A). The 1-μm wells had significantly more cells than the 350-nm gratings (*P* < 0.01, mean ± SD, *n* = 5), and similarly, the 250-nm wells had more cells than the 350-nm grating (*P* < 0.05, mean ± SD, *n* = 5). Comparing between the gratings, the 1-µm pattern has more attached cells than the 350-nm grating pattern, but the difference was not statistically significant.

As HMECs were cultured in serum-free medium, which might delay cell attachment, only a low number of cells had attached after 4 h. Even when using a more sensitive method in counting the DAPI-stained nuclei, low numbers of HMECs were attached on the patterned samples (Supplementary Figure S3), and a large variation was observed among replicas. Thus, the HMEC number was assessed 24 h after seeding with the enzymatic detachment method. The HMEC number after 24 h of culture on the various PLLA films (Figure 4B) showed that the 1-μm wells had fewer attached HMECs as compared to the 1-μm grating (*P* < 0.05, mean ± SD, *n* = 5). The highest number of HMEC adhered on the 1-μm gratings, while the lowest was noted in the 1-µm wells at the 24-hour time point.

To better mimic a heterogeneous mixture of mammary epithelial cells and cancer stem cell population in tumors, HMECs and MCF7 were co-cultured. Flow cytometry was performed on the co-culture to further investigate the adhesion properties of the two cell types with the objective of identifying the preferential adhesion of the breast cancer stem cells on different patterned PLLA. In the interpretation of the data, the HMEC was stained with CellTracker Red. The population of CellTracker Red-negative (MCF7) and CD44^+^ (mean ± SD, *n* = 3) in each sample was represented in the Figure 4C. The 350-nm gratings had a significantly higher percentage of CD44+ cells compared to the control (*P* < 0.05). For the 1-μm well, the percentage of CD44^+^ cells ranged from 0.06% to 26.4% with a standard deviation of 14.8%, which indicates a higher degree of inaccuracy or inconsistency in the readings. Meanwhile, the population of CellTracker Red-positive (HMEC) and CD44^+^ (mean ± SD, *n* = 3, Figure 4D) also showed that the 350-nm gratings and 1-µm gratings captured slightly higher percentages of CD44^+^ than the other samples, but no significant difference was observed among the samples.

### 2.4. Characterization of IDC Cells Adhered on Patterned PLLA Films

Invasive ductal carcinoma (IDC) cells, Passage 3–5, from breast tumor tissues of patient donors were obtained to investigate the preferential cell adhesion on different patterned PLLA films. To characterize the cells seeded on different patterns, cell counting, immunofluorescence staining and flow cytometry were employed to analyze the cell morphology and surface markers. 

**Figure 5:**
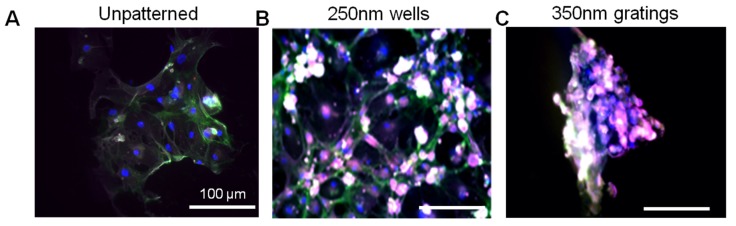
Immunofluorescence images of primary invasive ductal carcinoma (IDC) cells on (**A**) unpatterned PLLA film; (**B**) 250-nm wells and (**C**) 350-nm gratings after 4 h of culture. Blue: DAPI; green: CD44; red: CD24; magenta: ESA. Original magnification: 20×. Bar = 100 μm.

IDC cells attached well on the PLLA films 4 h after cell seeding in serum-free media. The IDC cells seeded on gratings were widely dispersed and had pronounced elongation and alignment in the direction of the gratings (Figure 5). The morphology of the primary cells (Figure 5) and the MCF7 cells (Figure 2) was very diverse. MCF7 cells cultured on the patterned films were flat with a spherical nucleus. By contrast, the primary cells showed stellate morphology, especially on the 250-nm wells and to a lesser extent on the 350-nm gratings.

As shown in the MCF7 immunostaining (Figure 2), heterogeneity was also observed in the immunophenotype of the primary cells (Figure 5) on the various patterned films after 4 h of culture. Surface marker expression varied across the population. Generally, most of the primary cells were observed to be CD44^+^, CD24^+^ and ESA^+^. Only minute numbers of them were CD44^+^CD24^−/low^ESA^+^. The preferential adhesion behavior of the CD44^+^CD24^−/low^ESA^+^ primary IDC cells on the nanoscale features was evident from the fluorescence images. 

**Figure 6:**
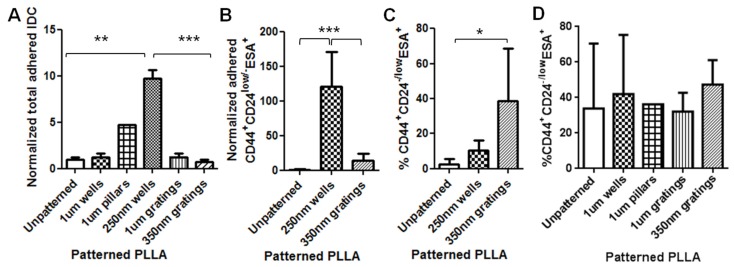
(**A**) Normalized number of IDC cells attached 4 h after seeding on PLLA films (**P < 0.01, ***P < 0.001, mean ± SD, *n* = 3; except for 1-µm pillars, where *n* = 1). The IDC cell adhesion behavior normalized to unpatterned control shows preference for nanoscale topography with the 250-nm wells. (**B**) The normalized number of primary IDC cells cultured after 4 h showed the 250-nm wells as the best topography to capture a significantly higher numbers of CD44^+^CD24^−/l^^ow^ESA^+^ cells (***P < 0.001, mean ± SD, *n* = 3). (**C**) By immunofluorescence staining and cell counting, the percentage of CD44^+^CD24^−/l^^ow^ESA^+^ showed that 350-nm gratings captured a significantly higher percentage of CD44^+^CD24^−/l^^ow^ESA^+^ cells (*P < 0.05, mean ± SD, *n* = 3). (**D**) Flow cytometry analysis of primary IDC cell culture after 4 h showed that 350-nm gratings captured a higher percentage of CD44^+^CD24^−/l^^ow^ ESA^+^ cells, but the difference was not significant (mean ± SD, *n* = 2; except for 1-µm pillars, where *n* = 1)

Due to the variations among patient samples, primary IDC cell counts varied significantly from one batch to another. The numbers of attached IDC cells on patterned samples were normalized to the number of IDC cells attached on the unpatterned control to facilitate the comparison over three different patient samples. Furthermore, due to the limited cell numbers available from each patient, the cell counting of the total cell number was performed using image analysis of the CellTracker Red- and DAPI-stained cells. From the cell counting, the 250-nm wells showed a significantly higher number of attached primary IDC cells, compared to the 350-nm gratings (*P* < 0.001) and unpatterned control (Figure 6A, *P* < 0.01). No significant difference in the normalized cell numbers was observed among the other patterns. 

Because both nanoscale patterns (350-nm gratings and 250-nm wells) captured a higher number of CD44^+^CD24^−/low^ESA^+^ cells from the MCF7 mono-culture at the 4-hour time point (Figure 3B), these two nano-patterns were chosen for cell counting of the immuno-stained population in the IDC cell experiment. The results showed that a significantly higher number of CD44^+^CD24^−/low^ESA^+^ cells were captured on the 250-nm well (Figure 6B). From the immunostaining observation, the number of CD44^+^CD24^−/low^ESA^+^ cells increased with the total cell population in the 250-nm wells; however, in terms of percentage, the CD44^+^CD24^−/low^ESA^+^ expression population was higher on gratings (Figure 6C). The 35-0nm gratings captured a significantly higher percentage of CD44^+^CD24^−/low^ESA^+^ compared to unpatterned control (*P* < 0.05). 

Flow cytometry was conducted twice on two batches of the IDC cells (Figure 6D). ANOVA (Kruskal–Wallis test followed by Dunn’s *post hoc* test) results did not show any significant difference among different patterned samples. However, a slightly higher percentage of CD44^+^CD24^−/low^ESA^+^ was observed on the 350-nm gratings, which agrees with our observations from counting immunofluorescently-stained samples (Figure 6C).

Comparing the total number of adhered cells and the CD44^+^ cells captured at 4-hour and 24-hour time points, the trends of the preferential adhesion of MCF7 were comparable at both time points. The different results between the IDC cell adhesion and MCF7 cell adhesion might be due to several factors. First, as the fluorescent staining required more washing steps, cells could have been washed away due to the varied strength of cell attachment to PLLA. Furthermore, the seeding densities used were different. Despite the discrepancy between both setups, we showed that MCF7, HMEC, IDC cells and the CD44^+^CD24^−/low^ESA^+^ cells among the cell populations have preferential attachment on different patterns. 

## 3. Discussion

Cancer stem cells (CSC) are a population of proliferating cells with the capability of self-renewal and re-establishment of tumor heterogeneity [22]. Since the establishment of the cancer stem cell theory, increasing evidence indicates that cancer stem cells play an important role in tumorigenesis and potential relapse of cancer. However, in general, current cancer therapies, such as chemotherapy, target rapidly proliferating cells instead of CSCs. As such, many have suggested that in order to reduce cancer relapse, eradication of CSCs is crucial. 

As cells are surrounded by submicron features in their microenvironment, it has been shown that nanotopography influences cell adhesion, proliferation and differentiation [23]. We hypothesize that different cell populations in breast cancer tissue exhibit different adhesion characteristics on substrates with nanotopography and investigated the adhesion characteristics of three cell populations, HMEC, breast cancer cell line MCF7 and primary IDC cells, on biomaterial substrates with nanotopographical cues. Based on the adhesion difference, the cancer stem cell-like population in MCF7, HMEC and IDC cells was separated and enriched from the mono-culture or the co-culture of HMEC and MCF7 using nanotopography and CSC surface markers. The results have shown a trend of preferential cell adhesion in both MCF7 and primary IDC cells cultured on different nanotopography after 4 and 24 h. Among the topographies tested, the nanoscale features of 350-nm gratings are consistently the most effective in enriching the CD44^+^CD24^−/low^ESA^+^ breast CSCs. Interestingly, the total cell population has preferential adhesion to 1-μm gratings and 250-nm wells for MCF7 and primary IDC cells, respectively, suggesting that sub-populations of cancer cells exhibit different adhesion characterization to topographical structures. 

Dick and colleagues’ studies in leukemic cells in the early 1990s gave one of the first descriptions of cancer stem cells [19]. They were able to isolate a subpopulation of leukemic cells via biomarkers CD34^+^/CD38^−^ and initiated acute myeloid leukemia in NOD/SCID mice using these leukemic cells [22,24]. CSCs were also found in the solid tumors of breast cancer [4,22,25,26]. Breast cancer cells have demonstrated not only self-renewal capability, but also a wide spectrum of progeny [25]. The ESA^+^CD44^+^CD24^−/l^^ow^ lineage cells isolated from human breast tumors were able to form tumors in eight-week-old NOD/SCID mice [4]. These cells formed a very small proportion of the total cell population, and as few as 200 cells were able to consistently form tumors in the mice and, thus, were identified as breast CSCs [4,25]. 

Although researchers were able to isolate these putative CSCs, a principle to isolate stem cells from any kind of cancer has yet to be established. In the case of breast cancer, there remains a need to determine whether CD44^+^CD24^−^ESA^+^ cells are true breast CSCs across various breast cancer subtypes. In addition, reports have confirmed that in both breast cancer-derived cell lines and breast tumors, CD44^+^CD24^−^ phenotypes were not necessarily associated with patient outcome or the ability to metastasize [7,27]. Therefore, further research is required to identify and separate CSCs in breast cancer. Meanwhile, current therapies fail to account for potential differences in drug sensitivity or target expression between the CSCs and the more frequent non-tumorigenic cells [28]. The ability to isolate breast CSCs enables the testing of the effectiveness of cancer drugs that are currently used and will also enable the development of therapeutic strategies aimed at selectively targeting breast CSCs [22].

Current methods for detecting and separating CSCs from solid tumors have been based on the observation of morphological features or labeling with specific markers [25]. The labeling-assisted method is more commonly used and is highly specific. However, it requires laborious procedures prior to separation and time-consuming post-processing, such as the removal of labels [29]. Label-free methods have been studied to separate cancer cells, which utilize differences in physical properties, such as cell size, density [20], cell adhesion [20,30] and dielectric properties [20], but the physical distribution is not high enough for efficient and accurate separation of cell populations, which limits their use. Kwon and co-workers used nanotopographic substrates to separate and enrich human breast cancer cells [19]. They demonstrated that normal human breast epithelial cells (MCF10A) have stronger adhesion to the substratum than MCF7, regardless of culture time, surface topography and flow rates. This separation technique was reported to have 78% efficiency [19]. 

Kwon’s study also found that the separation of human breast cancer cells from a mixed phase of MCF7 and MCF10A cells was optimized to occur after 2 h of culture on 400-nm gratings. A shorter duration of 2 h was chosen, as the high flow rate can exert significant shear damage to the adhered cells under longer culture periods. In contrast, in our static cultures, 4-hour is a reasonable duration, as the cells might not be able to recognize the surface within a short period of time. The 4-hour time point also appears to be the optimum duration for the separation and enrichment of the breast CSCs (as denoted by the phenotype of CD44^+^CD24^−/l^^ow^ESA^+^) from the heterogeneous MCF7 cancer cells and the primary cells under static culture conditions. This is in line with the development of a label-free method to isolate CSCs, as a shorter culture and analysis time is more desirable for cell analysis and cell isolation.

Kwon and colleagues determined that the use of 400-nm gratings resulted in the generation of the largest shear stress on the cell surface, facilitating the detachment of the MCF7 cells. Thus, it is the preferential detachment behavior that allowed the successful separation of the breast cancer cells in Kwon’s study [19]. On the contrary, we have focused on the preferential attachment behavior of the cells, which may account for the difference of the choice of topographical feature. Thus, future work may involve characterizing the unattached cells, as well, to establish the most effective nanotopography for the isolation of the CSCs. 

The mixture of MCF7 and MCF10A used by Kwon served to mimic the primary tissue of a breast cancer tumor; it has been proven that although breast and breast cancer cell lines have adapted to tissue culture conditions, they retain many of their phenotypic and genotypic properties over many passages. Hence, despite their acquired ability to grow *in vitro*, cell lines continue to share many of the molecular and genetic features of the primary breast cancers from which they were derived [8]. This validates the use of MCF7 to elucidate the preferential adhesion behavior of tumorigenic breast CSCs.

In this research, cell adhesion is evaluated based on the number of cells that adhered to the substrate and the cell morphology. From the data presented, less MCF7 and IDC cells adhered to gratings as compared to wells. From immunostaining, the MCF7 on the grating displayed higher fluorescence intensity for CD44 staining and a higher CD44^+^CD24^−/l^^ow^ESA^+^ population, as compared to wells and the control. By counting the number of CD44^+^ cells that adhered to the substrates, more than 40% of the cells that the 350-nm gratings captured were CD44^+^CD24^−/l^^ow^ESA^+^ MCF7 cells, suggesting that the 350-nm grating is most effective in the enrichment of CD44^+^CD24^−/l^^ow^ESA^+^ MCF7 in a monoculture. 

The difference in cell adhesion is a key parameter to enable isolation of breast CSCs from the heterogeneous cell suspension. Cell adhesion is affected by surface chemistry and the topography of the polymer substrate, as well as depending on the geometric parameters of the nanotopographic substrate. To mimic the heterogeneous cell population of a breast cancer tumor, the differential adhesion behavior of the co-culture of MCF7 and HMEC was studied. The nanotopography that would be the most suitable for the intended purpose would be one that captures the most CD44^+^ cells in the heterogeneous co-culture experiment. The MCF7 that adhered on 350-nm gratings continue to give high fluorescence intensity when stained for CD44, as compared to the well pattern, and have the highest percentage of cells that are CD44^+^ when analyzed by flow cytometry. This most likely indicates that in the co-culture, the nanotopography continues to affect the CD44^+^ population in MCF7 and HMEC by the same mechanism as when MCF7 was cultured alone, thus displaying a similar behavior.

Although the three different cell populations, MCF7, HMEC and IDC, showed different preferences in terms of the total cell adhesion on the 1-µm well, 1-µm gratings and 250-nm wells, respectively, the highest proportion of CD44^+^CD24^−/l^^ow^ESA^+^ was consistently captured on the 350-nm gratings. The observations supported our hypothesis that the different cell populations in the heterogeneous breast cancer tissue showed different preferential adhesion to various topographical patterns. In particular, the CD44^+^CD24^−/l^^ow^ESA^+^ CSC population showed a preferential retention and enrichment on 350-nm gratings, suggesting that this tumorigenic CSC population could be enriched by using nanoscaled gratings. However, it is uncertain whether the 350-nm grating induces the expression of CD44 or if it is able to isolate the cancer stem cell-like population in MCF7. This could be verified by studying whether the side population isolated by 350-nm gratings is indeed CSCs.

Cell adhesion is directly affected by cell-extracellular substrate interaction; however, the mechanism of the topographical regulation of cell adhesion remains unclear. One of the suggested mechanisms, known as “self-induced mechanotransduction” by Dalby *et al.*, is based on the hypothesis that surface topography alters nuclear morphology and chromosome position in adherent cells, leading to changes in gene transcription [31,32]. Another mechanism suggested by Ingber *et al.* is the “direct mechanotransduction” mechanism, whereby forces encountered by cells during cell adhesion are directly transmitted to nucleus via cytoskeleton, and altered cytoskeletal tension then feeds back to induce local changes in focal adhesion assembly [33,34]. Our group has demonstrated that topography regulates cell morphology by modulating the physical force-sensing of the cells [35,36]. Moreover, the focal adhesion signaling and cytoskeletal contractility are essential for nanotopographical regulation in stem cell differentiation [35,37]. 

Given the short time points (4 and 24 h) used in this research, it is unlikely that cell behavior would be affected by gene transcription. Preliminary data from cell adhesion counting and immunostaining support the concept that nanotopography affects cell behavior via a direct mechanism. Moreover, the morphology difference between the attached MCF7, HMEC and primary cells might provide a mechanistic explanation of the preferential cell adhesion. MCF7, HMEC and IDC cells showed elongated morphology on gratings patterns (Figure 2 and Figure 5); however, MCF7 and HMECs showed more pronounced cell spreading and elongation on gratings. Immunofluorescence staining of vinculin (Supplementary Figure S4) illustrated the focal adhesion of the MCF7 and HMECs on the patterns at 24 h. MCF7 on micro-sized patterns showed fewer focal adhesions, as compared to the nano-sized patterns, while HMECs showed well-established focal adhesion on all patterns. Further studies in cell morphology with SEM could be conducted to examine cell-topography interactions to provide a better understanding of the observed morphology differences. This will also illuminate the mechanism by which nanotopography influences cell adhesion. The cell morphology of the CD44^+^CD24^−^^/l^^o^^w^ESA^+^ cell should be further analyzed to provide a better basis for comparison of the cancer stem cell preferential adhesion among the different topographies.

In conclusion, between the two time points of four and 24 h, 4 h appear to be the optimum time mark for isolating the CD44^+^CD24^−/low^ESA^+^ tumorigenic cell population from both the MCF7 and the primary cell cultures. In the MCF7 and IDC and MCF-7-HMEC co-culture cell experiments, the trend of preferential adhesion behavior of these CD44^+^CD24^−/low^ESA^+^ cells on the nanoscale features was observed. Among the topographies tested, the 350-nm gratings are consistently the most efficient in enriching the CD44^+^CD24^−/low^ESA^+^ or breast CSCs from the MCF7 and primary IDC cell populations. Moving forward, to develop a biomaterial substrate for effective cancer stem cell isolation, the isolated cells can be further characterized and their cancer stem cell identity further studied, for instance, utilizing the ALDH1 enzyme as a potential marker for CSCs. Scaling up of the isolation and expansion of the isolated cells can also be further investigated. 

## 4. Conclusions 

We have successfully demonstrated the preferential adhesion of different breast cancer cell types on micro- and nano-sized topographies. From the adhesion test results, preferential cell adhesion of MCF7 and primary IDC cells cultured on different nanotopographies for 4 and 24 h was observed. The MCF7 and IDC cells are sensitive to the dimensions of the patterns as judged by a significant difference in total cell adhesion and the population of CD44^+^CD24^−/l^^o^^w^ESA^+^ captured. Among the topographies tested, the 350-nm grating is consistently most effective at inducing the attachment of a larger percentage of MCF7 and IDC cells with our proposed CSC phenotype CD44^+^CD24^−/l^^o^^w^ESA^+^. Flow cytometry analysis using two markers CD44^+^CD24^−/l^^o^^w^ also demonstrated that the 350-nm grating captured a larger number of CD44^+^CD24^−/l^^o^^w^ cells. For primary IDC cells, immunofluorescence analysis and flow cytometry analysis showed that the CD44^+^CD24^−/l^^o^^w^ESA^+^ cells had greater adherence on the 350-nm gratings. 

This study demonstrated that the subpopulations of cancer cells could exhibit different preferential adhesion behaviors. Therefore, by carefully selecting the optimal topography, the sub-group of cancer cells and CSCs could be separated by different topographies. 

## 5. Materials and Methods 

### 5.1. Fabrication of Micro- and Nano-Patterned Poly-L-Lactic Acid Films

A poly(dimethylsiloxane) (PDMS) soft mold was made from a micro- or nano-patterned template by soft lithography, as previously described [38]. Briefly, the PDMS 10:1 elastomer and curing agent mixture was degassed, dispensed onto the masters and cured at 65 °C.

PLLA films were made by solvent casting using 3% w/v PLLA (Sigma Aldrich, St. Louis, MO, USA) dissolved in chloroform on the PDMS soft mold. The films were dried at room temperature covered with a glass dish. PLLA films were vacuum dried in a desiccator for 5 min to remove any residual solvent before demolding. The PLLA films were trimmed to 1.0-cm^2^ squares and sterilized with 70% ethanol (v/v) and ultraviolet irradiation.

To verify the surface morphology and fidelity of the micro- and nano-topography replication process, PLLA films were sputter coated with gold (JEOL JFC 1600 Fine Gold Coater, JEOL Ltd., Tokyo, Japan) and examined by scanning electron microscopy (SEM, Quanta FEG 200 and JEOL JSM-5600LV Scanning Microscope, JEOL Ltd., Tokyo, Japan).

### 5.2. Cell Culture of Mammary Epithelial Cells, MCF7 and Primary Invasive Ductal Carcinoma Cells

HMEC (Lonza, Basel, Switzerland) were maintained using mammary epithelial basal medium (MEBM, Lonza) supplemented with bovine pituitary extract (BPE), human epithelial growth factor (hEGF), hydrocortisone, GA-1000 (Gentamicin/Amphotericin) and insulin. The medium was changed on alternate days. Cells were subcultured by washing with HEPES-buffered saline solution (HEPES-BSS), dissociated with 0.25× trypsin-EDTA and neutralized with trypsin-neutralizing solution (TNS) (Lonza). The human breast cancer cell line MCF7 was obtained from American Type Culture Collection (ATCC) and maintained using Dulbecco’s Modified Eagle’s Medium (DMEM, Sigma Aldrich, St. Louis, MO, USA) supplemented with 10% fetal bovine serum (FBS), 0.01 mg/mL bovine insulin, 1% penicillin streptomycin and 1% non-essential amino acid solution. The medium was changed every three days. Invasive ductal carcinoma (IDC) cells (Passage 3–5) from breast tumor tissues of patient donors were obtained from the NUH-NUS (Singapore) Tissue Repository with NUS Institutional Review Board approval (08-090). They were maintained using the mammary epithelial growth medium (MEGM, Lonza) supplemented with 10% FBS, amphotericin B and gentamicin. 

Cells were trypsinized upon reaching 80% confluence and seeded on PLLA films with a seeding density of 10,000 cells/cm^2^ (MCF7) or 16,000 cells/cm^2^ (HMEC). The substrates were then placed in an incubator for static culture. In the co-culture of MCF7 and HMEC, 16,000 cells/cm^2^ of each cell type (HMEC and MCF7) were seeded onto each of the samples and the control. The cell culture was maintained using MEGM. To differentiate both cell types in the co-culture system, HMEC was stained with CellTracker™ Red CMPTX (Invitrogen, Waltham, MA, USA) by incubating with 10 µM of the dye in serum-free medium (MEGM) for 30 min and subsequently replaced with MEGM for another 30 min.

### 5.3. Cell Adhesion Counting

The number of attached cells on nanotopographic substrates in monocultures was determined after 4 and 24 h. Unattached cells were washed twice with PBS or HBSS. Cells were then detached with trypsin from the PLLA films, and the cell number was determined by hemocytometer counting. Adhesion assays were performed with 5 replicates. 

In another method, cell adhesion counting was performed after 4 h of culture. In order to identify the live cells, the attached cells were stained with the dye, CellTracker™ Red CMPTX (Invitrogen). After 3 h of culture, cells were labeled with CellTracker dye. At the 4-hour time point, the samples were gently washed to remove the unattached cells, then fixed with 4% formaldehyde solution and stained with DAPI. The samples were washed and mounted before imaging with a fluorescent microscope (Leica DC 300F, Leica, Wetzlar, Germany). The number of adhered cells was determined by counting the DAPI- and CellTracker-stained cells. For each sample, 5 random images were taken at 20× magnification. Cell adhesion counting was carried out for both HMEC and MCF7 mono-cultures.

### 5.4. Immunostaining of CD44, CD24 and Epithelial Surface Antigen Markers

After 4 or 24 h of culture, the cells were fixed with 4% formaldehyde and incubated in blocking buffer (1% w/v bovine serum albumin (BSA) and 10% goat serum in PBS). The cells were stained with antibodies specific for human cell surface markers: CD44-FITC (Abcam, Cambridge, UK), CD24-PE (Abcam) and ESA (Santa Cruz, Starr County, TX, USA) (Table 1). After overnight incubation at 4 °C, the cells were washed and incubated with the corresponding secondary antibodies: Alexa Fluor 488, Alexa Fluor 546 and Alexa Fluor 647 (Invitrogen) for 1 h at room temperature. The cells were counter-stained with DAPI. For each sample, 5 random images were taken at 20× magnification. The number of cells that were of the CD44^+^CD24^−^ESA^+^ phenotype was counted. The CD24^−/low^ immunophenotype was taken as CD24^−^ for cell counting.

**Table 1:** Dilution ratio of the antibodies used in immunostaining.

Antigen	Primary antibody	Secondary antibody
CD44	Anti-CD44, rat, conjugated with FITC 1:500	FITC, anti-rat 1:750
CD24	Anti-CD24, mouse, conjugated with PE 1:50	Alexa Fluor 546, anti-mouse 1:750
ESA	Anti-ESA, rabbit, unconjugated 1:50	Alexa Fluor 647, anti-rabbit 1:750

### 5.5. Flow Cytometry Analysis

To analyze the expression of CD44, CD24 and ESA after 4 or 24 h of culture, flow cytometry was performed on the MCF7 mono-culture and IDC cell experiment. At the 4- or 24-hour time point, the cells attached on the films were trypsinized, centrifuged and counted. The harvested cells were incubated with 2% goat serum in staining buffer (1% goat serum, 0.5% BSA in PBS) for 10 min in an ice bath and stained with antibodies specific for human cell surface markers: CD44-FITC (Abcam), CD24-PE (Abcam) and ESA (Santa Cruz) (Table 2). After incubation for 30 minutes at 4 °C, the suspensions were washed three times in staining buffer. Cells were then incubated with the secondary antibody, Alexa Fluor 647 for ESA, for 30 min at 4 °C. The cells were washed twice and fixed with 1% formaldehyde for 1 h before analyzing with the flow cytometer (Dako Cytomation Cyan LX, Glostrup, Denmark). The data were analyzed by FlowJo (version 10.0.7).

**Table 2:** Dilution ratio of the antibodies used in flow cytometry.

Antigen	Primary antibody	Secondary antibody
CD44	Rat anti-CD44 conjugated with FITC 1:50	None
CD24	Mouse anti-CD24 conjugated with PE 1:10	None
ESA	Rabbit anti-ESA Un-conjugated 1:100	Goat anti-rabbit conjugated with Alexa Fluor 647 1:500

### 5.6. Statistics Analysis

Data are presented as the mean ± SD unless specified otherwise. The statistical significance of the results obtained was assessed by one-way analysis of variance (ANOVA) and Tukey’s *post hoc* test or the Kruskal–Wallis test followed by Dunn’s *post hoc* test for flow cytometry analysis data. Results with *P* < 0.05 were considered statistically different. 

## Supplementary Files

Supplementary File 1
