# Supplementary material for: Differential Cell Adhesion of Breast Cancer Stem Cells on Biomaterial Substrate with Nanotopographical Cues
Source: J Funct Biomater. 2015 Apr 21;6(2):241–58. doi: 10.3390/jfb6020241 (PMC4493510; doi:10.3390/jfb6020241)
Supplement: Supplementary File 1 [file jfb-06-00241-s001.pdf]

OPEN ACCESS
Journal of
Functional Biomaterials
ISSN 2079-4983
www.mdpi.com/journal/jfb

Supplementary Materials

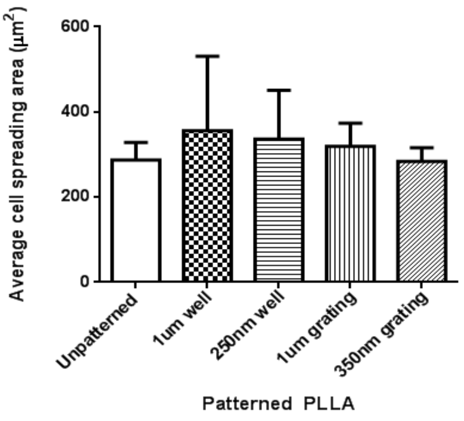

Figure S1. Cell spreading area of MCF7 on the PLLA samples.

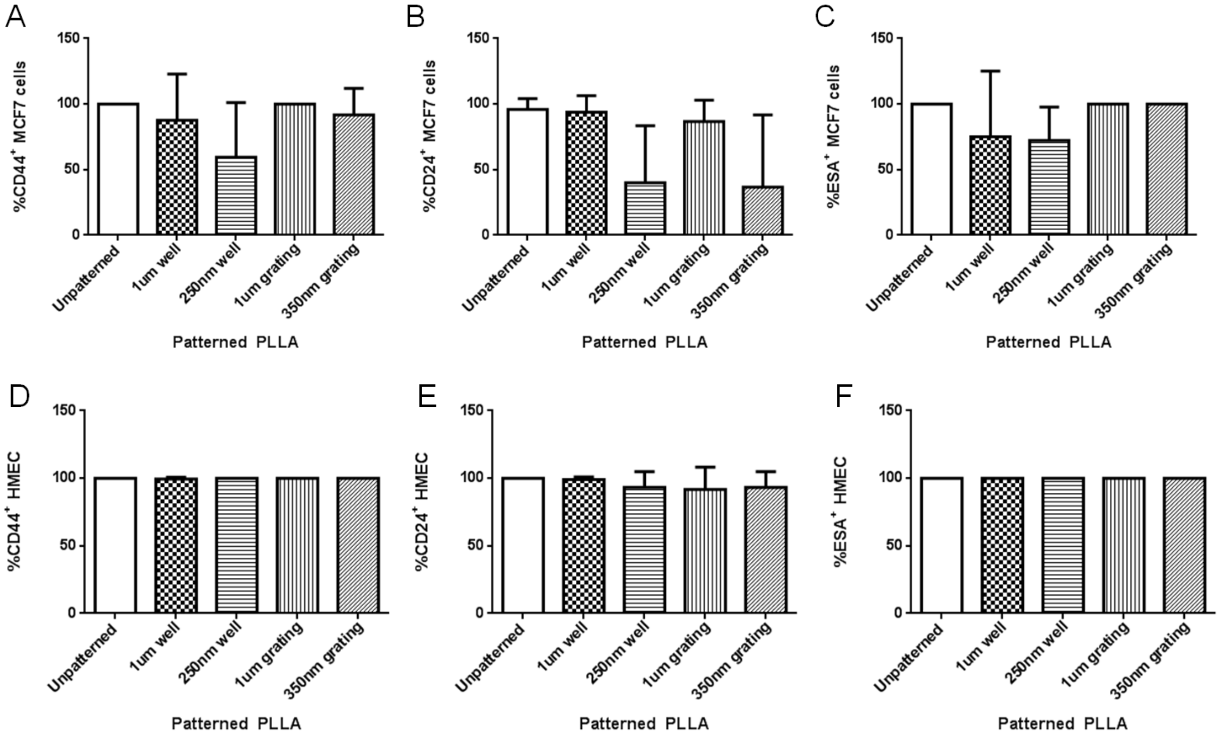

Figure S2. Percentage of MCF7 cells expressing CD44 (**A**), CD24 (**B**), and ESA (**C**) on different patterns. Percentage of HMECs expressing CD44 (**D**), CD24 (**E**), and ESA (**F**) on different patterns.

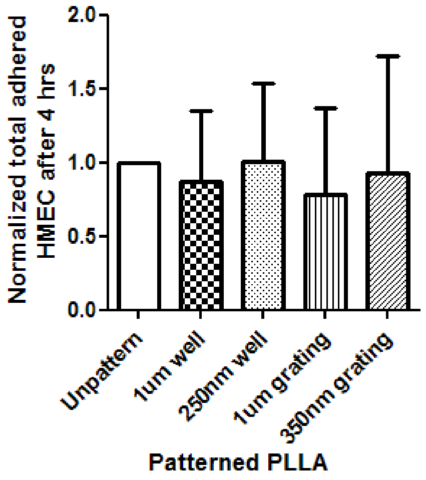

Figure S3. Number of HMEC attached 4 h after seeding on PLLA films, seeding density 16,000 cells/cm^2 No significant difference was observed among patterned samples. (mean ± SD, n = 4).

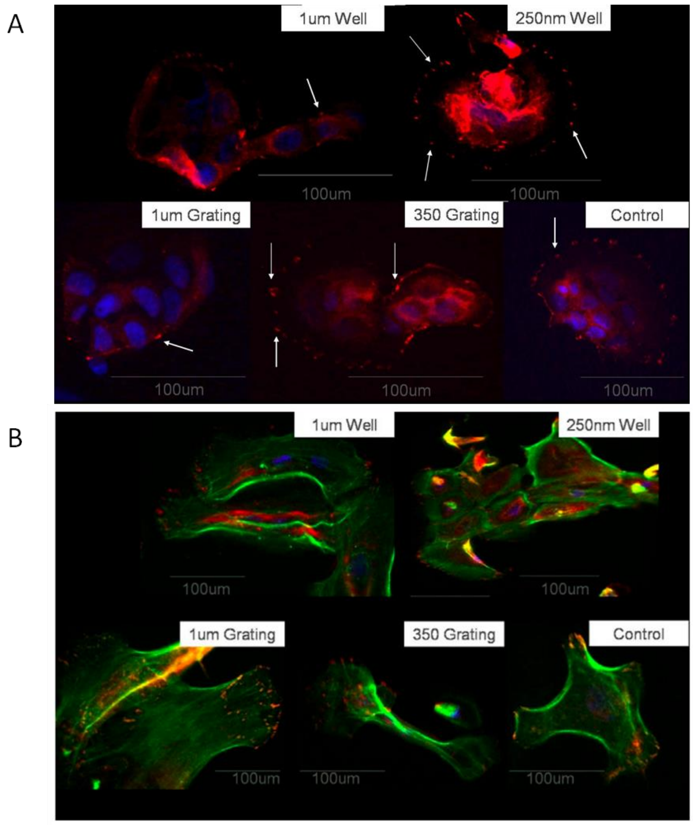

Figure S4. (**A**) Immunofluorescence images of vinculin (red) and DAPI (blue) in MCF7 after 24 h of culture. MCF7 seeded on the micro-sized patterns show significantly fewer focal adhesion complexes as compared to the nano-sized patterns. As compared to control, the 250 nm well and 350 gratings show similar number of focal adhesions. (**B**) Immunofluorescence images of F-Actin (green), vinculin (red) and DAPI (blue) in HMEC after 24 h of culture. HMEC seeded on grating are very spread out and they have pronounced elongation; the cells also aligned themselves along the directions of the grating. Bar = 100 μm.
